# Influence of Observers’ Motor Imagery Abilities on Observational Gait Analysis Using the Wisconsin Gait Scale: A Study Among Physical Therapists Treating Stroke Patients

**DOI:** 10.7759/cureus.82163

**Published:** 2025-04-13

**Authors:** Takayuki Maru, Shun Sawai, Shoya Fujikawa, Ryosuke Yamamoto, Takato Nishida, Yusuke Shizuka, Hideki Nakano

**Affiliations:** 1 Department of Physical Therapy, Faculty of Health Sciences, Kyoto Tachibana University, Kyoto, JPN; 2 Department of Rehabilitation, Junshin Kobe Hospital, Kobe, JPN; 3 Graduate School of Health Sciences, Kyoto Tachibana University, Kyoto, JPN; 4 Department of Rehabilitation, Kyoto Kuno Hospital, Kyoto, JPN; 5 Department of Rehabilitation, Tesseika Neurosurgical Hospital, Kyoto, JPN; 6 Department of Physical Therapy, Faculty of Rehabilitation and Care, Seijoh University, Tokai, JPN

**Keywords:** controllability of motor imagery test, observational gait analysis, physical therapist, stroke, wisconsin gait scale

## Abstract

Observational gait analysis is a simple gait assessment commonly used in rehabilitation. However, the effect of an observer’s experience and ability on gait analysis has not been clarified. This study aimed to describe observers’ motor imagery ability using the Wisconsin Gait Scale (WGS), an evaluation index for observational gait analysis in patients with stroke. Thirty-two physical therapists participated in this study. All participants observed a gait video of a patient with a stroke and performed observational gait analysis using the WGS. The number of correct answers to the WGS for each participant was then calculated according to the correct answers created by two physical therapists with experience in treating patients with stroke. In addition, we evaluated the participants’ motor imagery ability using the Controllability of Motor Imagery Test (CMI-T). Multiple regression analysis was performed to examine the factors related to the number of correct WGS answers, with the dependent variable being the number of correct WGS answers and the independent variables being years of experience and CMI-T scores. Next, a hierarchical cluster analysis was performed using the number of correct WGS answers and years of experience as variables, and the CMI-T scores were compared between the two clusters. The results showed that the number of years of experience was selected as a factor significantly related to the number of correct WGS answers. Clusters with more correct WGS answers and more years of experience had substantially higher CMI-T scores than those with fewer correct answers and less experience. In conclusion, physical therapists with better observational gait analysis and more years of experience have better motor imagery skills than their counterparts.

## Introduction

Many patients with stroke present with gait disturbances that significantly impair their mobility in daily life [1]. Gait autonomy is directly related to the ability of patients with stroke to lead independent lives. Therefore, gait evaluation and intervention are important in stroke rehabilitation. However, the gait of patients with stroke varies because of the various symptoms that occur depending on the brain regions affected by the stroke. Specifically, many temporally and spatially complex abnormal gait patterns are observed, such as asymmetrical gait, extension thrust pattern with excessive knee joint extension during the stance phase, and circumduction gait with toe clearance by hip abduction during the swing phase [2,3]. Therefore, it is necessary to evaluate and treat abnormal gait patterns in each individual patient. In addition, abnormal gait patterns in patients with stroke are not only directly attributable to functional decline due to stroke but also include those caused by compensating for functional decline in other parts of the body [1,4]. Therefore, abnormal gait patterns in patients with stroke are complex, and intervention is important after an accurate assessment.

In many cases, the gait of patients with stroke is evaluated by observational gait analysis by rehabilitation professionals, such as physical therapists [5]. An objective evaluation technique using three-dimensional (3D) motion analysis has recently been developed [6], but it is costly and time-consuming. Furthermore, free software using markerless motion capture has been designed to solve this problem, but its accuracy is low [7]. These factors have led to the widespread use of observational gait analysis in stroke rehabilitation compared with objective gait analysis techniques. Physical therapists need accurate observational gait analysis. Many studies have developed assessment tools to accurately evaluate abnormal gait patterns in patients with stroke [8,9]. The Wisconsin Gait Scale (WGS) is an evaluation index that assesses the gait of patients with stroke both temporally and spatially [10]. The WGS can improve the accuracy of observational gait analysis by evaluating gait according to the gait cycle and body parts [11]. In addition, the WGS is related to other gait parameters, such as 3D gait analysis and gait speed [12,13], and has moderate construct validity for gait, balance, and functional measures in patients with acute stroke [14]. Thus, it has been suggested that using the WGS may improve the accuracy of observational gait analysis for patients with stroke. However, although the WGS assists in improving the accuracy of observational gait analysis of patients with stroke by itemizing the points to be focused on to evaluate the presence or absence of abnormal gait patterns accurately, it is necessary for the physical therapist who evaluates the patients to have a high level of observational gait analysis ability. Thus, physical therapists must perform accurate observational gait analysis, and evaluation indices, such as the WGS, have been developed to support this.

Motor imagery is the cognitive process of imagining that one is exercising without actually performing the movement or tensing the muscles [15]. It improves motor performance even when no movement is being performed and is used in motor skill acquisition and rehabilitation [16]. Motor imagery is a mental activity that involves the internal generation of the visual and kinesthetic aspects of movement. In contrast, behavioral observations, such as observational gait analysis, involve mental processes that evoke internal motor representations of the observed movement [17,18]. Thus, behavioral observations and motor imagery, such as observational gait analysis, have demonstrated similar mental activities. Since both motor imagery and behavioral observation involve mental simulations of movement, an observer's motor imagery ability may influence the accuracy of observational gait analysis. Furthermore, motor imagery and motor execution share neural activity, indicating that motor-related brain regions are involved in both processes [19]. Furthermore, motor imagery shares some neural activity with behavioral observations, such as observational gait analysis [20]. In particular, the premotor-parietal and somatosensory networks are commonly activated during motor imagery and action observation [21]. As described above, action observation and motor imagery, such as observational gait analysis, are similar in that they mentally rehearse movements and share common neural substrates. Notably, the premotor cortex has been reported to be associated with movement observation and may remarkably influence visual-motor imagery [22]. Thus, the accuracy of observational gait analysis may be related to motor imagery ability. However, the relationship between observed gait analysis and motor imagery remains unclear due to the limited number of studies. Clarifying the role of motor imagery in observational gait analysis may allow for the examination of teaching methods aimed at improving physical therapists' skills.

This study focused on motor imagery ability and clinical experience as factors affecting the accuracy of observational gait analysis. In particular, we examined the following research questions: (i) Is motor imagery ability associated with the accuracy of observational gait analysis? (ii) How does clinical experience affect the ability to perform an accurate gait assessment? Therefore, this study aims to address these research gaps by focusing on the following specific objectives: (1) Evaluate the relationship between motor imagery ability and the accuracy of observational gait analysis. (2) Determine the influence of clinical experience on observational gait analysis performance. (3) Compare motor imagery ability between groups with high versus low observational accuracy. We hypothesized that physical therapists with higher accuracy in observational gait analysis would have better motor imagery ability than their counterparts.

## Materials and methods

Participants

The participants in this study were 32 physical therapists (22 males and 10 females; mean age, 29.50 ± 8.20 years) from a medical institution that primarily provides rehabilitation mainly for cerebrovascular diseases. Each participant had between 0.25 and 16.5 years of clinical experience at the time of data collection (months were converted to years). They worked in the acute and sub-acute wards of a single medical facility. Those with experience in rehabilitating stroke patients featured in the WGS video observation were excluded from this study.

Ethics declarations

This study was conducted in accordance with the Declaration of Helsinki and was approved by the Ethical Review Committee of Junshin Kobe Hospital (approval number: 003). Informed consent was obtained from all the participants. We informed the participants that the study aimed to evaluate the accuracy of observational gait analysis and did not disclose the detailed hypotheses.

Observational gait analysis evaluation

The intent of this study was to evaluate gait analysis in a manner similar to observational gait analysis in a clinical setting; however, concerns about patient fatigue affecting gait led to the use of video-based analysis. The WGS was used to evaluate observational gait analysis. It is an observational tool used to evaluate gait quality in patients with stroke hemiplegia. The WGS is divided into four subscales, with 14 items evaluating the temporal and spatial parameters of gait observed during the gait cycle [10]. The following gait characteristics were evaluated based on the WGS. The stance phase of the affected leg was assessed by considering the use of a hand-held gait aid, stance time on the impaired side, step length of the unaffected side, weight shift to the affected side (with or without a gait aid), and stance width (measured as the distance between feet prior to toe-off of the affected foot). In the toe-off phase of the affected leg, we evaluated whether the patient exhibited guardedness, which refers to a pause before advancing the affected leg, as well as a hip extension, by observing the gluteal crease from behind. The swing phase of the affected leg was analyzed in terms of external rotation during an initial swing, circumduction at mid-swing by observing the path of the affected heel, hip hiking at mid-swing, knee flexion from toe-off to mid-swing, toe clearance, and pelvic rotation at terminal swing. Finally, during the heel strike of the affected leg, the nature of initial foot contact was examined. Item 1 was scored on a five-point scale, item 11 on a four-point scale, and the other items on a three-point scale. Items 1 and 4 were weighted 3/5 and 3/4, respectively, and each item was scored on a 42-point scale.

Observational gait analysis using the WGS was performed using a walking movie of an 82-year-old man with stroke hemiplegia. The patient with stroke hemiplegia had a Brunnstrom stage [23] of five upper limbs, five lower limbs, five fingers, and good cognitive function. Indoor walking was independent without a cane, and outdoor walking was independent with a T-cane. Two tablet terminals (iPad, Apple Inc., USA) were used to capture the patient’s gait. Video recordings were made only once after providing physical therapy and a well-deserved break. The video frame rate was 23.98 frames per second. The tablets were positioned 80 cm above the floor, 3 m lateral to the center of the walking path, and 1 m behind the walking path. The patient’s gait was filmed from two directions, corresponding to the sagittal and frontal planes. The walking path was 13 m long, including an extra 1.5 m at both the beginning and the end, and the patient walked at a comfortable speed using a T-cane.

Before the observational gait analysis using the WGS, each participant received a thorough explanation of the WGS and its evaluation method and was then instructed to observe the patient’s walking videos. While watching the videos, participants rated the 14 items on the WGS without pausing, rewinding, or taking notes. A laptop computer (ProBook 450 G5, HP Inc., Palo Alto, CA) running Windows Media Player (Microsoft Corp., USA) was used to play the videos. The walking videos were played 10 times consecutively, and the WGS evaluation forms were collected 10 minutes after the start of video playback. Two male physical therapists, with 11.67 and 15.58 years of experience, respectively, observed the patient’s gait videos. Both were frequently treated stroke patients. The number of correct answers in the WGS for each participant was calculated based on the predetermined correct answers. The creation of the WGS solution involved the two experienced physical therapists viewing and discussing the video to formulate the correct solution. In case of disagreement, they discussed the evaluation details in depth. In addition, one researcher evaluated all participants in the WGS.

Assessment of motor imagery ability

**Figure 1:**
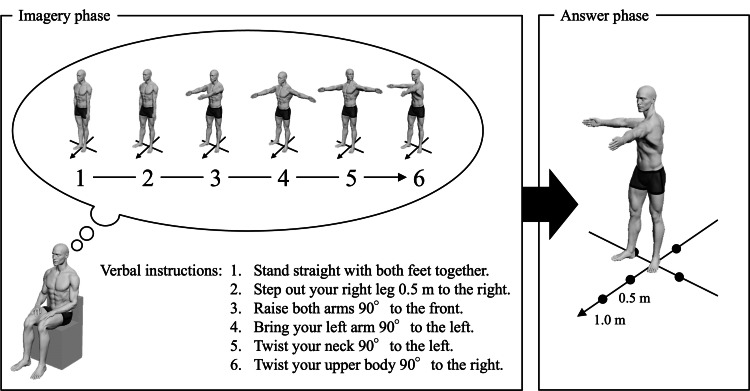
Example of the Controllability of Motor Imagery Test (CMI-T) The CMI-T consisted of imagery and answer phases. In the imagery phase, the participant sat on a chair with eyes closed and was instructed to visualize postures and body part movements according to a series of six verbal instructions. After the last verbal instruction, the participants stood with their eyes open and reproduced the final posture that came to mind during the answer phase. Image credits: Takayuki Maru, Shun Sawai, and Hideki Nakano

The Controllability of Motor Imagery Test (CMI-T) was used to assess motor imagery ability. It was selected because it was expected to detect relatively high motor imagery ability in healthy adults and requires the reproduction of imagined postural changes. This replication makes it a more objective measure than questionnaire-based assessments. The CMI-T was administered as previously described [24,25]. The CMI-T consisted of 10 questions, and the order of the questions was the same for all participants. The CMI-T was administered to the participants after a thorough explanation of the CMI-T and its administration. The CMI-T consisted of imagery and answer phases (Figure 1). The imagery phase consisted of six consecutive verbal instructions regarding posture and body movements. The first instruction was for the basic posture, and the next five instructions were for body part movements (left upper or right upper limb, left lower or right lower limb, trunk, or neck).

These six consecutive verbal instructions differed for all 10 questions. The interval between each verbal instruction was approximately two seconds, and the participant was presented with a prerecorded voice. The participant was seated in a chair with eyes closed and instructed to visualize the posture and body part movements according to six consecutive verbal instructions.

After the last verbal instruction, the participants stood with their eyes open and reproduced the final posture that came to mind during the answer phase. On the floor, marked lines indicate the direction in 45° increments and the distance from the center (0.5 m and 1.0 m). Using these indicators, the participant was instructed to accurately reproduce the final posture (orientation, position, and distance of each body part). If the participant could not answer the entire final posture, he was allowed to reproduce only the part that he could answer. The final posture during the answer phase was captured using a digital video camera from the forehead, and sagittal planes, and its correctness was determined through offline analysis. In this analysis, points were awarded for the correct final posture (rated by orientation, position, and distance) of each body part (left or right arm, left or right leg, trunk, and neck) for six consecutive verbal instructions with reference to an image of the correct final posture for each trial according to a standardized procedure. For each question, correct answers were counted (six points each), and the CMI-T score was calculated by summing the correct answers across 10 questions (totaling 60 points). In addition, one researcher evaluated all participants in the CMI-T.

Statistical analyses

First, the Shapiro-Wilk test was used to confirm the normality of all data. Next, a multiple regression analysis using the stepwise variable reduction method was performed to examine the factors related to the number of correct WGS answers. The independent variables were years of experience and CMI-T scores, and the dependent variable was the number of correct WGS answers. Then, hierarchical cluster analysis was performed using the number of correct WGS answers and years of experience as variables. In the cluster analysis, the two clustering variables were standardized using Z-scores. The Ward method was used for the clustering method, and the distance between individuals was measured using the Euclidean squared distance. The chi-squared test was used to compare the sex among the clusters. Unpaired t-tests or Mann-Whitney U tests were used to compare age, years of experience, number of correct WGS answers, and CMI-T scores among the clusters. SPSS version 29.0.2.0 (IBM SPSS Statistics for Windows, IBM Corp., Armonk, NY) was used for statistical analyses. The significance level was set at <5%.

## Results

**Figure 2:**
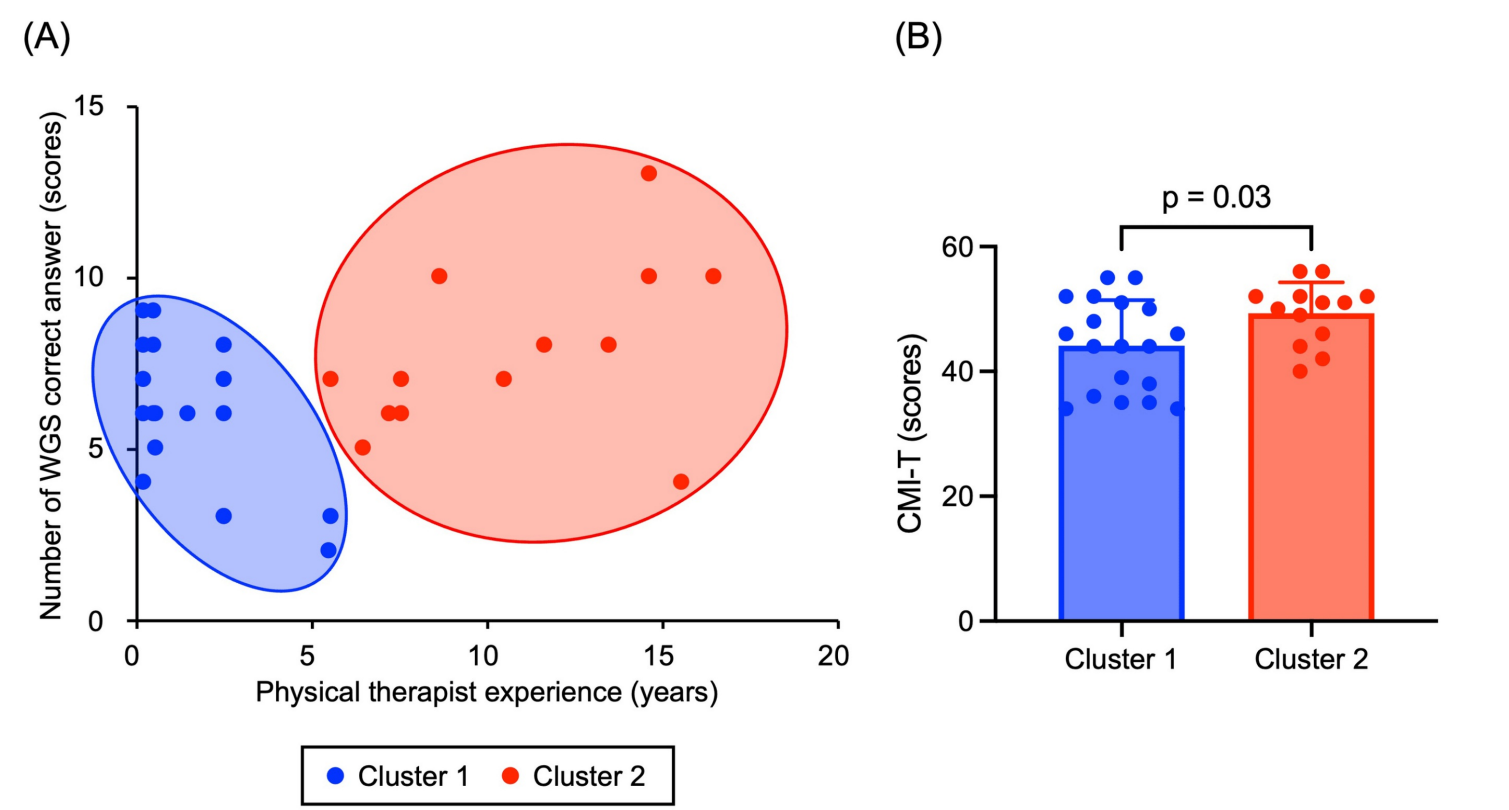
Results of the hierarchical cluster analysis and comparison of the CMI-T between clusters (A) The results show two subgroups based on the number of correct WGS answers and years of experience. Blue circles indicate Cluster 1, and red circles indicate Cluster 2. (B) Cluster 2 had significantly higher CMI-T scores than Cluster 1 (p < 0.05). CMI-T, Controllability of Motor Imagery Test; WGS, Wisconsin Gait Scale

The demographics of the participants are summarized in Table 1. Multiple regression analysis revealed that years of experience were selected as the factor that significantly affected the number of correct WGS answers (β = 0.36, p = 0.04, Table 2), suggesting that clinical experience contributes to the accuracy of observational gait analysis. Hierarchical cluster analysis classified the number of correct WGS answers and years of experience into two subgroups: Clusters 1 and 2 (Figure 2A). Cluster 2, which had a higher number of correct WGS answers, also had significantly more years of clinical experience than Cluster 1 (p < 0.05). This finding suggests that observational gait analysis skills improve with increased experience. The chi-squared test result showed no significant differences between the clusters in terms of sex (p = 0.11). In contrast, Cluster 2 had significantly more years of experience, a significantly higher age, a higher number of correct WGS answers, and higher CMI-T scores than those exhibited by Cluster 1 (p < 0.05, Table 3, Figure 2B). This result implies that motor imagery ability may be indirectly associated with observational gait analysis accuracy through its relationship with clinical experience.

**Table 1:** Participant demographics Data are presented as means ± standard deviations or numbers. CMI-T, Controllability of Motor Imagery Test; WGS, Wisconsin Gait Scale *Months were converted to years.

	All participants (n = 32)
Age (years)	29.5 ± 8.20
Sex, male/female (n)	22/10
Physical therapist experience (years^*^)	5.30 ± 5.36
Current primary practice setting, acute/sub-acute (n)	11/21
Number of correct WGS answers (scores)	6.66 ± 2.40
CMI-T (scores)	46.22 ± 6.89

**Table 2:** Results of the multiple regression analysis The p-value was calculated using multiple regression analysis with the variable reduction method. The t-value and p-value correspond to the coefficient significance test for the predictor variable in the regression model. VIF, variance inflation factor; WGS, Wisconsin Gait Scale

Model	Independent variables	β	t-value	p-value	95% CI		VIF
					Lower	Upper	
1	CMI-T	0.15	0.79	0.44	-0.08	0.18	1.18
	Physical therapist experience	0.30	1.63	0.11	-0.03	0.31	1.18
2	Physical therapist experience	0.36	2.12	0.04	0.01	0.32	1.00
-	Adjusted R^2^	0.10	-	-	-	-	-

**Table 3:** Comparison of characteristics of the participants between Cluster 1 and Cluster 2 Data are presented as means ± standard deviations or numbers. The test statistic is presented as the Mann-Whitney U value for the Mann-Whitney U test, the chi-square value for the chi-square test, and the t-value for the t-test. p-value was calculated using the Mann-Whitney U test for age, physical therapist experience, and CMI-T; the chi-squared test for sex and current primary practice setting; and the unpaired t-test for the number of correct WGS answers. CMI-T, Controllability of Motor Imagery Test; WGS, Wisconsin Gait Scale *Months were converted to years.

	Cluster 1 (n = 19)	Cluster 2 (n = 13)	Test statistic	p-value
Age (years)	24.42 ± 3.95	36.92 ± 7.04	239.00	<0.01
Sex, male/female (n)	11 / 8	11 / 2	2.57	0.11
Physical therapist experience (years^*^)	1.54 ± 1.70	10.79 ± 3.84	246.50	<0.01
Current primary practice setting, acute/sub-acute (n)	6 / 13	5 / 8	0.16	0.69
Number of correct WGS answers (scores)	5.90 ± 2.11	7.77 ± 2.46	-2.31	0.03
CMI-T (scores)	44.11 ± 7.33	49.31 ± 4.97	178.50	0.03

## Discussion

This study aimed to examine the effect of motor imagery on the accuracy of observational gait analysis performed by physical therapists. First, multiple regression analysis indicated that years of physical therapist experience were significantly related to the number of co-WGS answers. Next, hierarchical cluster classification was conducted using the number of correct WGS answers and years of experience. The CMI-T scores of the two clustered groups were compared. The results showed that the CMI-T scores of the group with the highest number of correct WGS answers and years of experience were significantly higher than those of the group with the lowest number of correct WGS answers and years of experience. The results of this study suggest that motor imagery ability may be related to the accuracy of observational gait analysis.

Multiple regression analysis revealed that years of experience as a physical therapist were significantly related to the number of correct WGS answers. Previous studies have not reached a certain conclusion, with some reporting that experience does not affect observational skills in gait analysis [26], whereas others have reported that more specialized and skilled physical therapists can perform accurate observational gait analysis [27]. According to Bonkohara [26], experience with gait analysis does not affect the ability to identify changes in joint motion per se; rather, it influences the ability to recognize and judge joint motion as deviating from normal. Since the WGS used in this study consists of an ordinal scale [10] and physical therapists are responsible for determining whether gait deviates from normal, it is possible that years of experience affected the number of correct answers on the WGS. These findings suggest that the years of experience of physical therapists may be related to the accuracy of observational gait analysis.

In this study, hierarchical cluster classification was conducted using the number of correct WGS answers and years of experience as variables, and the participants were divided into two groups. The results showed that Cluster 1 had significantly fewer years of experience and a significantly lower number of correct WGS answers than Cluster 2. This indicates that the accuracy of the physical therapists' observational gait analysis was not consistent across observers and that there was variability in its accuracy. Observational gait analysis [28] and videotaped gait observation [29] are less accurate and have slight to moderate reliability. Similar to previous studies, the results of this study indicate that the accuracy of observational gait analysis performed by physical therapists is not consistently high. Furthermore, this study compared the CMI-T scores between a group with a low number of correct WGS answers and years of experience (Cluster 1) and a group with a high number of correct WGS answers and years of experience (Cluster 2). The results showed that Cluster 2 had a significantly higher CMI-T score than Cluster 1. This indicates that physical therapists with more years of experience who perform accurate observational gait analyses have better motor imagery abilities. Spatial and temporal parameters of gait after stroke are significantly different from those of healthy participants [30].

Furthermore, the symptoms and gait of patients with stroke are complex and vary among individuals [2,3]; thus, physical therapists need to have a high spatial cognitive ability to perform accurate observational gait analysis. In the CMI-T, which was used in this study as an assessment item for motor imagery ability, it is necessary to mentally generate, manipulate, and hold imaginary postures through trials. Spatial cognitive ability is necessary for mental stimulation while performing tasks [26]. Because both observational gait analysis and motor imagery require the mental simulation of movements and spatial cognitive functions, it is possible that physical therapists with higher accuracy in observational gait analysis have higher motor imagery ability. In addition, previous studies on neural activity in the brain have shown that when observing and trying to understand the actions of others, the parietal lobes are activated from the premotor cortex [31]. The neural networks of action observation and motor imagery, including the premotor-parietal and somatosensory networks, are similar [21]. As described above, physical therapists with higher accuracy in observational gait analysis may have higher motor imagery abilities because of the similarity in the characteristics of neural activity between behavioral observation and motor imagery, such as observational gait analysis. In addition to these findings, physical therapists with more experience can perform observational gait analyses on many patients in clinical situations. Although this has not yet been verified in previous studies, we speculate that this experience may improve motor imagery ability for gait movements by repeatedly imagining the observed gait movements in the brain using spatial cognitive abilities. A previous study has demonstrated that individuals with higher performance exhibit greater motor imagery ability compared to those with lower performance [20]. Therefore, repeated observation and motor imagery of gait by physical therapists during clinical treatment may enhance the accuracy of observational gait analysis. In summary, this study suggests that physical therapists' years of experience are an important factor influencing the accuracy of observational gait analysis. However, due to the cross-sectional nature of this study, causal relationships cannot be concluded, and these findings should be considered preliminary. Our results suggest that developing motor imagery skills alongside clinical experience may improve the reliability of observational gait analysis, although whether one directly influences the other requires further study. Moreover, while motor imagery ability appears relevant, its precise influence requires further investigation. Prior research has reported that skilled clinicians tend to perform more vertical exploration in eye movements, which may contribute to their proficiency in observational gait analysis [26]. Furthermore, visual pattern recognition and memory may play a role in rapidly detecting gait abnormalities. Therefore, future studies should assess attentional, visual, and spatial cognitive abilities in addition to motor imagery ability to examine their relationship to the accuracy of observational gait analysis. This comprehensive approach would enhance the accuracy of clinical assessments. The participant population in this study consisted primarily of relatively inexperienced physical therapists, which allowed us to identify challenges in performing observational gait analysis in a clinical setting. The results could potentially inform the design of education and training programs for young physical therapists. In general, the primary goal of observational gait analysis is simply to observe gait. However, our findings suggest that gait analysis should capture gait not only visually but also temporally and spatially within the context of motion imagery and that repeated application of this process may further improve analysis accuracy. Furthermore, previous research has demonstrated that motor imagery and behavioral observation share overlapping neurologically relevant networks [21] and that combining them can further enhance motor performance [32]. In the present study, we found that motor imagery is relevant to gait analysis. Although additional validation is needed, given that this is a cross-sectional study, our results suggest that combining motor imagery training with observational gait analysis may effectively enhance performance.

This study has some limitations that should be considered when interpreting the results. First, this was a cross-sectional study, so causal relationships between the accuracy of observational gait analysis and either motor imagery or years of experience could not be established. Although feedback is ineffective in improving motor imagery ability, the method for training motor imagery remains unclear [16]. To enhance the accuracy of observational gait analysis in future studies, effective intervention methods should be examined using longitudinal validation. Second, the sample size of this study was relatively small, and the participant population consisted mainly of inexperienced physical therapists, which may limit the generalizability of the results. Future surveys should include physical therapists with more diverse clinical experience to improve generalizability. Moreover, the preliminary explanation provided to participants outlined the general purpose of the study without specifying hypotheses regarding the relationship between motor imagery ability and observed gait analysis. While complete blinding was not feasible, we attempted to minimize potential biases by ensuring that participants were unaware of the study's specific research focus. Nevertheless, the possibility of implicit bias cannot be entirely ruled out. Future studies should implement more rigorous blinding procedures, such as multiple task conditions or partial disclosure, to further reduce potential expectancy effects. Third, the correct answers for the observational gait analysis were determined through consultation between two experienced physiotherapists, so an objective evaluation could not be conducted. The WGS solution was developed by having two experienced physical therapists watch and discuss the video to formulate the correct answers, but no inter-rater reliability test was performed. For a more accurate gait analysis, it is necessary to measure the joint angles in each gait using a 3D motion analyzer. Additionally, WGS scoring should be validated with objective measures and inter-rater reliability tests. Future studies should determine the accuracy of observational gait analysis by creating correct answers based on objective indices. Finally, the cases included in the observational gait analysis exhibited relatively mild motor paralysis, which may affect the generalizability of the findings to cases with severe disabilities. We also did not collect information on participants' educational background or training experience in gait analysis, which may be a confounding factor. Future studies should gather more detailed background information for a more rigorous analysis.

## Conclusions

This study investigated the role of motor imagery abilities in observational gait analysis (using the WGS) among physical therapists evaluating patients with stroke. Our findings highlight that physical therapists with more years of experience tend to perform more accurate gait assessments, and importantly, those with higher observational accuracy also demonstrate superior motor imagery abilities. This suggests that motor imagery may facilitate the effective analysis of gait patterns. Given that observational gait analysis remains a widely used method in clinical practice due to its simplicity and accessibility, it is essential to understand the cognitive components that influence its accuracy. The results of this study indicate that fostering motor imagery abilities, in addition to clinical experience, could enhance the reliability of observational gait analysis. Future studies should explore intervention strategies to improve motor imagery abilities in physical therapists and examine whether such training leads to enhanced assessment accuracy in real-world clinical settings. Additionally, longitudinal studies are needed to determine the causal relationship between motor imagery ability and the accuracy of gait assessment.
